# Early experience of aortic surgery during the COVID‐19 pandemic in the UK: A multicentre study

**DOI:** 10.1111/jocs.15307

**Published:** 2021-01-13

**Authors:** Ana Lopez‐Marco, Amer Harky, Danilo Verdichizzo, Emma Hope, Barbara Rosser, Iain McPherson, Ronan Kelly, Luke Holland, Aung Ye Oo, The UK AS Research Group

**Affiliations:** ^1^ Department of Cardiothoracic Surgery Barts Heart Centre, St. Bartholomew's Hospital London UK; ^2^ Department of Cardiothoracic Surgery Liverpool Heart and Chest Hospital Liverpool UK; ^3^ Department of Integrative Biology, Faculty of Health and Life Science University of Liverpool Liverpool UK; ^4^ Liverpool Centre for Cardiovascular Science University of Liverpool and Liverpool Heart and Chest Hospital Liverpool UK; ^5^ Department of Congenital Cardiac Surgery Alder Hey Children Hospital Liverpool UK; ^6^ Department of Cardiothoracic Surgery John Radcliffe Hospital Oxford UK; ^7^ Department of Cardiothoracic Surgery University Hospital of Southampton Southampton UK; ^8^ Department of Cardiothoracic Surgery Royal Brompton and Harefield NHS Trust London UK; ^9^ Department of Cardiothoracic Surgery Freeman Hospital Newcastle UK; ^10^ Department of Cardiothoracic Surgery Royal Victoria Hospital Belfast UK; ^11^ Department of Cardiothoracic Surgery Royal Sussex County Hospital Brighton UK

**Keywords:** aorta, aortic dissection, aortic surgery, pandemic

## Abstract

**Background:**

A significant restructuring of the healthcare services has taken place since the declaration of the coronavirus disease 2019 (COVID‐19) pandemic, with elective surgery put on hold to concentrate intensive care resources to treat COVID‐19 as well as to protect patients who are waiting for relatively low risk surgery from exposure to potentially infected hospital environment.

**Methods:**

Multicentre study, with 19 participating centers, to define the impact of the pandemic on the provision of aortovascular services and patients' outcomes after having adapted the thresholds for intervention to guarantee access to treatment for emergency and urgent conditions. Retrospective analysis of prospectively collected data, including all patients with aortovascular conditions admitted for surgical or conservative treatment from the 1st March to the 20th May 2020.

**Results:**

A total of 189 patients were analyzed, and 182 underwent surgery. Diagnosis included: aneurysm (45%), acute aortic syndrome (44%), pseudoaneurysm (4%), aortic valve endocarditis (4%), and other (3%). Timing for surgery was: emergency (40%), urgent (34%), or elective (26%). In‐hospital mortality was 12%. Thirteen patients were diagnosed with COVID‐19 during the peri‐operative period, and this subgroup was not associated with a higher mortality.

**Conclusions:**

There was a significant change in service provision for aortovascular patients in the UK. Although the emergency and urgent surgical activity were maintained, elective treatment was minimal during early months of the pandemic.

The preoperative COVID‐19 screening protocol, combined with self‐isolation and shielding, contributed to the low incidence of COVID‐19 in our series and a mortality similar to that of pre‐pandemic outcomes.

**Abbreviatons**
CT: computed tomography
NHS: National Health Service
PEARS: personalized external aortic root support
PPE: personal protective equipment
UK: United Kingdom

## 1 INTRODUCTION

Severe acute respiratory syndrome coronavirus 2, the virus that causes coronavirus disease 2019 (COVID‐19), was first described a as cluster of cases of pneumonia at Wuhan (China) on the 31 December 2019.^1^ The number of cases exponentially increased and spread rapidly to other geographical locations, reaching the status of a global pandemic on the 11 March 2020.^2^

An extensive restructuring of the healthcare services has taken place due to the need for reallocation of intensive care resources to treat patients with COVID‐19. In the United Kingdom (UK) elective surgery was put on hold during the early months of the pandemic to concentrate resources on acute services as well as to protect surgical patients from exposure to potentially infected hospital environments.^3^

At the beginning of the lockdown in the UK, we redefined the current guidelines for treatment of aortovascular pathologies and adapted the thresholds for intervention to the current service provision.^4^ A multicentre service evaluation study was designed to assess the effects of the COVID‐19 pandemic on the delivery of services and clinical outcomes of the aortovascular patients. In this article, we report the initial experience in the UK during the early months of the pandemic.

## 2 METHODS

Retrospective analysis of prospectively collected data from all patients who were admitted with aortovascular pathologies (aortic root, ascending aorta, arch, descending thoracic aorta, and/or thoracoabdominal aorta) during the early months of the pandemic (1 of March to 20 May 2020) in the 19 participating centers from the UK.

The participating centers, grouped by regions, are: London—Barts Heart Centre, Royal Brompton and Harefield Hospitals, and Hammersmith Hospital; Southeast—Royal Sussex County Hospital (Brighton), University Hospital Southampton, and John Radcliffe Hospital (Oxford); West Midlands—Queen Elizabeth Hospital (Birmingham), University Hospital Coventry, and Royal Stoke University Hospital; East Midlands—Glenfield Hospital (Leicester); Northwest—Liverpool Heart and Chest Hospital and Blackpool Victoria Hospital; Yorkshire and Humberside—Sheffield Teaching Hospital and Castle Hill Hospital (Hull); Northeast—Freeman Hospital (Newcastle) and James Cook University Hospital (Middlesbrough), Scotland—Royal Infirmary of Edinburgh and Aberdeen Royal Infirmary, and Northern Ireland—Royal Victoria Hospital (Belfast).

This centers represent 66% of the aortic units in the country, including the largest specialized aortic centers, and cover most of the geographical areas.

The other 15 aortic units declined to participate in the study for different reasons including inability to provide emergency surgey cover during the COVID‐19, insufficient resources to collect the data and/or individual preferences.

Details about preoperative demographics and risk factors, operative details, and postoperative complications were obtained from National Cardiac Databases of the individual centers. A detailed analysis of the COVID‐19 screening peri‐operatively and its effect on decision making and timing of the treatment were also analyzed.

The anonymized patient data from individual centers were transferred securely to St. Bartholomew's Hospital for data cleaning and analysis. Data analysis was performed with SPSS version 25, including descriptive analysis of numeric (mean and range) and categorical values (total number and percentages) as well as *p* values when comparing different groups according timing for surgery (*χ* ^2^ test).

Ethical approval was obtained from each participating center after acceptance of the study protocol at the recruiting center (St. Bartholomew's Hospital). Individual patient consent was waived due the anonymised nature of the data.

### 2.1 Service provision for emergency and urgent aortovascular conditions

A protocol to treat aortovascular pathologies was created at Barts Heart Centre on the 25 March 2020 and endorsed by the UK Aortic Surgery group and the Society of Cardiothoracic Surgery for Great Britain and Ireland (SCTS).^5^, ^6^, ^7^, ^8^

The protocol defines the cohort of aortovascular patients eligible for referral and treatment during the COVID‐19 pandemic, triaged in several categories depending on the level of urgency at time of referral/presentation.

- Level 1: Elective—asymptomatic patients with indications for routine surgery should be added to an elective waiting list and be reviewed regularly with the plan to treat when the COVID‐19 pandemic resolves.
- Level 2: Urgent—the revised agreed thresholds for intervention during the COVID‐19 pandemic, after the appropriate screening, included: large aneurysms (> 6 cm for root, ascending and arch [> 5.5 cm for confirmed genetic aortopathies], >6.5 cm for descending thoracic aorta, and >7 cm for abdominal aorta), patients with new or persistent chest/back pain and other urgent conditions such as pseudoaneurysms, mycotic aneurysms and aortic graft infections.
- Level 3: Emergency—patients with acute aortic syndromes (type A aortic dissection, intramural hematoma, penetrating aortic ulcer, aortic transection, and acute complicated type B aortic dissection) and ruptured aneurysms of any anatomical location should be accepted and operated at the earliest opportunity, including out‐of‐hours, due to the increased risk of mortality while waiting.

### 2.2 Preoperative COVID‐19 screening

On the 26 March 2020 a preoperative screening protocol for detection of SAVR‐CoV‐2 virus was intorudces at St. Bartholomew's Hospital after multidisciplinary review of available evidence an Public Health Guidance analysis.^3^, ^9^, ^10^

It included a combination of two negative nasophayngeal swabs for polymerase change raction for ribonucleic acid (PCR‐RNA) analysis, a noncontrast computed tomography (CT) thorax to assess changes in the lung parenchyma suggestive of COVID‐19 disease and analysis of the lactate dehydrogenase levels and lymphocyte counts.^9^, ^10^

These results were not awaited in emergency situations (i.e., acute De Bakey I aortic dissection)

Patients who tested positive in the preoperative screening posed a challenge and were managed in isolation within highly‐specialized clinical areas, monitoring their symptoms while waiting for the COVID‐19 infection to resolve, if and when time permitting.

Irrespective of the patients COVID‐19 status, the surgical procedures and postoperative care in intensive therapy unit (ITU) environments were performed using universal measures of personal protective equipment (PPE).^11^

## 3 RESULTS

A total of 182 patients with aortovascular pathologies were operated from 1 March to 20 May 2020 in the 19 participating centers.

Mean age was 63 years (range 26–83 years), 33% of the patients were female and the mean EuroScore II was 9.6 (range 0.9–61.2). The details of preoperative risk factors are listed in Table 1.

**Table 1:** Demographics and preoperative risks factors

	Total	Elective	Urgent	Emergency	
	*n* (%)/mean (range)	*n* (%)/mean (range)	*n* (%)/mean (range)	*n* (%)/mean (range)	*p*
Age	63 (26–83)	63 (26–84)	61 (26–82)	61 (27–85)	.51
Female sex	60 (33%)	20 (43.5%)	17 (28.3%)	23 (30.2%)	.22
Hypertension	132 (72.5%)	31 (67.4%)	46 (76.7%)	55 (72.4%)	.48
Diabetes	14 (7.7%)	6 (13%)	4 (6.7%)	4 (5.3%)	.32
Dyslipidemia	30 (16.5%)	10 (22%)	10 (16.7%)	10 (13.1%)	.46
COPD	18 (9.9.%)	7 (15.2%)	6 (10%)	5 (6.6%)	.30
Creatinine	97.7 (42–864)	97.0 (45–109)	97.1 (59–864)	97.7 (42–288)	.71
Dialysis	2 (1.1%)	0	1 (1.7%)	1 (1.3%)	.69
Ex‐smoker	49 (26.9%)	20 (43.5%)	20 (33.4%)	9 (11.8%)	.001
Current smoker	27 (14.8%)	5 (10.9%)	7 (11.7%)	15 (19.7%)	.41
Previous stroke	8 (4.4%)	7 (15.2%)	0	1 (1.3%)	.57
Previous TIA	7 (3.8%)	3 (6.5%)	1 (1.7%)	3 (3.9%)	.72
Peripheral vascular disease	10 (5.5%)	2 (4.3%)	3 (5%)	5 (6.6%)	.68
Prior myocardial infarction	10 (5.5.%)	1 (2.2%)	5 (8.4%)	4 (5.3%)	.37
Prior PCI	2 (1.1%)		1 (1.7%)	1 (1.3%)	.69
Poor EF	5 (2.7%)	1 (2.2%)	1 (1.7%)	4 (5.3%)	.05
Moderate EF	26 (14.3%)	4 (8.7%)	15 (25%)	7 (9.2%)	.01
Atrial fibrillation	19 (10.4%)	4 (8.7%)	8 (13.4%)	7 (9.2%)	.35
Prior cardiac surgery	27 (14.8%)	6 (13%)	15 (25%)	6 (7.9%)	.02
Prior aortic surgery	23 (12.6%)	7 (15.2%)	12 (20%)	4 (5.3%)	.03
Prior endovascular treatment	7 (3.85)	2 (4.3%)	2 (3.4%)	3 (3.9%)	.55
EuroScore II	9.6 (0.9–61.2)	9.6 (0.9–20.3)	9.6 (1.9–61.2)	9.6 (1.9–42.7)	.40

Abbreviations: COPD, chronic obstructive pulmonary disease; EF, ejection fraction; PCI, percutaneous coronary intervention; TIA, transient ischemic attack.

The aortovascular pathologies mandating admission during the pandemic period were: aneurysm (82 patients, 45%), aortic dissection (71 patients, 39%), intramural hematoma (7 patients, 3.8%), penetrating aortic ulcer (2 patients, 1.1%), pseudoaneurysm (7 patients, 3.8%), aortic valve endocarditis (7 patients, 3.8%), and other (5 patients, 2.7%).

Timing for surgical intervention was considered as emergency (76 patients, 42%), urgent (60 patients, 33%), or elective (46 patients, 25%).

Anatomical location of the aneurysms and the mean aortic diameters were as follows: aortic root (*n* = 31, mean size 44 mm (range 31–86 mm); ascending aorta (*n* = 56, mean size 47 mm (37–90 mm); aortic arch (*n* = 5, mean size 47 mm (40–53 mm); descending thoracic aorta (*n* = 9, mean size 60 mm (55–100 mm), and thoracoabdominal aorta (*n* = 5, mean size 55 mm (51–60 mm).

The acute aortic syndromes were categorized depending on antomical presentation and chronicity as follow: acute BeBakey I (*n* = 61), acute DeBakey II (*n* = 5), acute DeBakey III (*n* = 5), chronic DeBakey I (*n* = 1), and chronic DeBakey III (*n* = 3).

The type of surgical procedures performed were: aortic root replacement (*n* = 55), valve‐sparing root replacement (*n* = 11), aortic root plasty (*n* = 5), personalized external aortic root support (PEARS) procedure (*n* = 1), aortic valve and ascending aorta replacement (*n* = 41), isolated ascending aorta replacement (*n* = 29), arch replacement (*n* = 24), frozen elephant trunk repair (*n* = 9), descending thoracic aorta replacement (*n* = 13), thoraco‐abdominal aorta replacement (*n* = 4), and endovascular procedures (*n* = 6).

Overall in‐hospital mortality was 12.1% (22 patients), with a 5.5% rate of intra‐operative deaths (10 patients). Causes of death were listed as followed: Cardiac related (*n* = 11), multiorgan failure (*n* = 5), neurological (*n* = 3), mesenteric ischemia (*n* = 2), and other (*n* = 1).

Postoperative complications included reintubation (4.4%) and tracheostomy (7.1%), postoperative stroke (8.8%), and need for hemofiltration (8.8%). Mean intubation and ventilation time was 2.9 days (30 min–53 days) and mean length of ITU stay was 4.7 days (30 min–53 days; Table 2).

**Table 2:** Postoperative complications

	Total	Elective	Urgent	Emergency	
	*n* (%)/Mean (range)	*n* (%)/Mean (range)	*n* (%)/Mean (range)	*n* (%)/Mean (range)	*p*
Intraoperative death	10 (5.5%)	0	0	10 (13.1%)	.001
In‐hospital death	22 (12.1%)	0	2 (3.4%)	20 (26.3%)	.001
Length ITU stay (h)	112.3 (0.5–1272)	115.6 (1–600)	115.3 (1–440)	115.6 (2–1272)	.18
Ventilatory time (h)	71.5 (0.5–1272)	76.3 (3–150)	73.6 (2–440)	74.1 (0.5–1272)	.24
Reintubation	8 (4.4%)	3 (6.5%)	0	5 (6.6.%)	.13
Tracheostomy	13 (7.1%)	2 (4.3%)	3 (5%)	8 (10.5%)	.21
Reoperation for bleeding/tamponade	14 (7.7%)	3 (6.5%)	3 (5%)	8 (10.5%)	.47
GI bleeding	2 (1.1%)	0	2 (3.4%)	0	.12
Mesenteric ischemia	3 (1.6%)	0	1 (1.7%)	2 (2.6%)	.54
Stroke	16 (8.8%)	2 (4.3%)	3 (5%)	11 (14.5%)	.04
Myocardial infarction	1 (0.5%)	1 (2.2.%)	0	0	.23
Spinal cord injury	1 (0.5%)	1 (2.2.%)	0	0	.36
Renal failure	25 (13.7%)	2 (4.3%)	7 (11.2%)	16 (21.1%)	.03
Haemofilter	16 (8.8.%)	1 (2.2%)	4 (6.7%)	11 (14.5%)	.05
Atrial fibrillation	47 (25.8%)	10 (21.7%)	13 (21.7%)	24 (31.6%)	.34
Sternal wound infection	6 (3.3.%)	2 (4.3%)	4 (6.7%)	0	.05

Abbreviations: GI, gastrointestinal; ITU, intensive therapy unit.

In‐hospital mortality was significantly higher, and almost exclusive, in patients presenting as emergency as opposed to elective procedures (*p* = .001). Other postoperative complications such as stroke, renal failure and haemofiltration and sternal wound infection were also significantly higher in the emergency group (*p* < .005; Table 2).

### 3.1 Patients who did not receive surgery during the COVID‐19 pandemic

There were six patients who were not operated after assessment in specialized aortovascular units.

Five patients presented with acute type A aortic dissections and the decision for not offering them emergency surgical treatment was based on clinical complexity (three patients had history of previous cardiac or aortic surgery) and comorbidities rather than COVID‐19 disease (only patient had CT changes suggestive of COVID‐19). The surgical turn down rate was 2.6% and mortality in this group was 60%.

Three patients with a type A aortic dissection died due to aortic rupture before surgery could be offered (en‐route to the surgical center or in the anesthetic induction).

One patient presented with uncomplicated acute DeBakey III aortic dissection and did not require surgical treatment. He was diagnosed of COVID‐19 during the hospital admission but did not develop any symptoms or respiratory complications.

### 3.2 COVID‐19 screening

Preoperative COVID‐19 status was unknown in 135 patients (74.1%), either because it was not performed (most of the regions were not performing routine tests before the 25th March) or because the results were not awaited due the critical emergency nature of the diagnosis.

Forty three patients (24.7%) were confirmed COVID‐19 negative before their operation and four patients (2.2%) were diagnosed of COVID‐19 during the preoprative period and their operation delayed until they became swab negative.

Preoperative CT chest for assessment of COVID‐19‐related changes in the lung parenchyma was only available in 86 patients (47.2%). Only two patients showed suggestive changes of COVID‐19 in the CT, and in one of them they were already present in the preoperative CT but the severity of his aortic condition overweighted the risk of developing COVID‐19 related complications and surgical indication was made.

### 3.3 Confirmed COVID‐19 disease

A total of 13 patients were diagnosed of COVID‐19 disease in the peri‐operative period (7.1%).

Seven patients (3.8%) were diagnosed in the postoperative period (mean postoperative Day 11 (range 2–24). Of them only one required prolonged mechanical ventilation but there was no associated mortality.

Four patients (2.2%) were diagnosed during the preoperative screening and their operations were delayed until COVID test confirmed negative (mean delay 9 days [range 2–13 days]). None of these patients developed any COVID‐19 related complications.

One patient had signs of COVID‐19 lung disease on the preoperative CT scan despite being asymptomatic and swab negative. He required reintubation and ventilation 7 days after surgery due chest infection and died of respiratory failure.

The last patient, mentioned earlier, was treated conservatively for acute DeBakey III aortic dissection and did not develop any COVID‐19 related complications.

### 3.4 Temporal and regional variation in delivery of aortovascular services

There was a clear temporal and regional variation on the delivery of aortovascular services.

**Figure 1:**
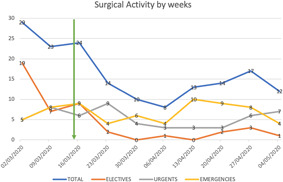
Temporal varitation in the delivery of aortovascular services. Surgical activity displayed weekly including total number of cases (blue line) and grouped by timing of the operation: elective (orange line), urgent (gray line), and/or emergency (yellow line). The vertical green arrow marks the start of the lockdown situation in the UK. There was a clear reduction of surgical activity after the start fo the lockdown, with almost disappearance of the elective surgical activity during the first months of the pandemic in the UK

The reduction of surgical activity was noticeable coinciding with the start of the lockdown in the UK on the 23th March, and reflected the interruption of elective surgery in London after the second week of the pandemic. Similarly, the number of elective operations decreased significantly in the rest of the regions in UK. However, from weeks 5 to 7 onwards some elective activities restarting slowly in certain regions (South East, North West and Yorkshire, and Humberside), while in the majority of areas the elective activity was completely stopped during the first months of the pandemic (Figure 1).

**Figure 2:**
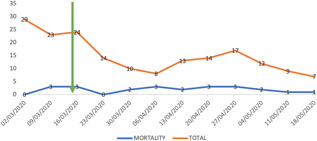
Temporal variation in the mortality of patients with aortovacular conditions treated in the participating centers in the UK over the COVID‐19 pandemic period. The vertical green arrow marks the start of the lockdown situation in the UK. The blue line displays weekly mortality from aortovascular conditions compared to the the total number of aortovacualr conditions treated in the same period in the participating centers (orange line). Note that the mortality trend for aortovascular conditions was constant during the early months of the pandemic in the UK

The number of urgent and emergency operations has been slowly increasing weekly, reaching a plateau by Week 9 (end of April). The regions providing the largest nonelective activity were London, North West and South East (Figures 1 and 2).

Other regions such as West Midlands have seen their aortovascular activity almost interrupted due to a more strict restructuring of services coinciding with a higher incidence of COVID‐19 patients in the areas covered (Figure 2).

The London region lead the provision of services due to the creation of the Pan London Emergency Cardiac Surgery Service, that concentrated all the surgical activity for the region into two of the service delivery centers (Barts Heart Centre and Brompton and Harefield NHS Trust) designed as COVID‐19 free‐environments (Figure 2).

**Figure 3:**
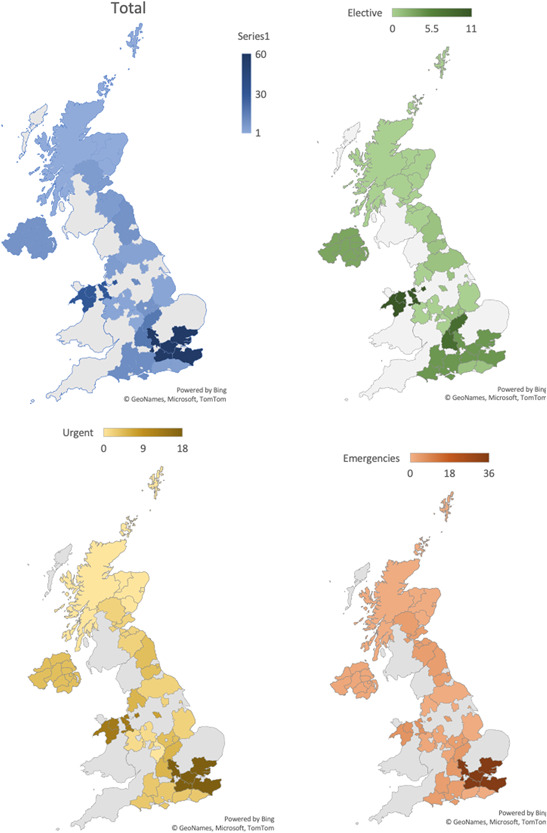
Geographical variation in the presentation of aortovascular conditions to hospital during the study period in the 19 participating centers in the UK. The different UK maps display the overall admissions (blue) as well as per level of emergency: elective cases (green), urgent cases (yellow), and emergency cases (orange). The graded colors represent the number of patients with aortovascular ondictions admitted to hospital for assessment and/or surgical treatment according each geographical region. The areas displayed in gray were the regions covered by centers not contributing to the study

**Figure 4:**
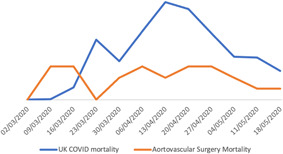
Graph showing the trend in mortality due to coronavirus disease 2019 (COVID‐19) in the UK displayed weekly (blue line) and the surgical aortovascular mortality during the same period of time in the study participating centers (orange line). The lines cross‐over on the week of the 16 March 2020, corresponding with the start of the lockdown in the UK, when the number of COVID‐19 cases started to increase exponentially and the Aortovascular activity decreased initially due to the reduced presentation to hospitals. Both curves reached a peak around mid of April to descend in a parallel way after that. Note that he scale for the COVID‐19 mortality has been adapted and has to be multiplied ×100

The majority of regions have managed to maintain the emergency activity when compared with the pre‐pandemic period, as obtained by comparing the acute aortic syndromes treated in the equivalent months last year prior the pandemic (Table 3). Mortality due aortovascular conditions treated during this period was similar to national benchmarked results from pre‐pandemic times^12^ and the trend was maintained during the early months of the pandemic (Figures 3, 4).

**Table 3:** Number of patients with acute aortic syndromes operated in each of the participating centers during the study period (pandemic activity) and during the equivalents months prior the pandemic (March–May 2019; pre‐pandemic activity)

Centre	Pandemic activity	Pre‐pandemic activity
St. Bartholomew's Hospital	27 (34.2%)	14 (17.5%)
Royal Brompton and Harefield NHS Trust	8 (10.1%)	6 (7.5%)
Hammersmith Hospital	0	3 (3.7%)
Royal Sussex County Hospital	1 (1.3%)	5 (6.2%)
University Hospital Southampton	6 (7.6%)	5 (6.2%)
John Radcliffe Hospital	6 (7.6%)	6 (7.5%)
Queen Elizabeth Hospital	0	3 (3.7%)
University Hospital Coventry	1 (1.3%)	2 (2.5%)
Royal Stoke University Hospital	1 (1.3%)	0
Glenfield Hospital	2 (2.6%)	1 (1.2%)
Liverpool Heart and Chest Hospital	7 (8.9%)	9 (11.2%)
Blackpool Victoria Hospital	4 (5.1%)	2 (2.5%)
Sheffield Teaching Hospital	6 (7.6%)	3 (3.7%)
Castle Hill Hospital	2 (2.6%)	2 (2.5%)
Freeman Hospital	5 (6.3%)	4 (5%)
James Cook University Hospital	1 (1.3%)	2 (2.5%)
Royal Infirmary of Edinburgh	3 (3.4%)	4 (5%)
Aberdeen Royal Infirmary	0	1 (1.2%)
Royal Victoria Hospital Belfast	3 (3.4%)	4 (5%)

## 4 DISCUSSION

In the UK, like in the rest of the world, the COVID‐19 Pandemic affected the healthcare service provision significantly. The National Health Service (NHS) was re‐organized across all countries in UK with the guidance of the Public Health England and the Central Government. Private healthcare providers were also included in the overall provision of service across every regions.

There was a clear reduction on the surgical activity in all units coinciding with the start of the lockdown on the 23th March, reaching the lowest activity by the third week of the lockdown to increase slowly afterwards, mainly due to the protection of the emergency services.

Elective aortovascular surgical activity discontinued almost completely after the lockdown status was declared and it has only been restarted very slowly in certain regions of the UK towards the end of April 2020, while in the majority of the regions has been completely stopped (Figure 1).

The opposite effect has been seen with the emergency services; the combined urgent and emergency activity decreased coinciding with the lockdown announcement but increased progressively during each week of April (Figure 1).

The reduction of elective surgery, which came to halt in most of the regions, is explained by the restructuration of the healthcare systems, preserving the critical care capacity to attend COVID‐19 patients who would require respiratory and multiorgan support, as well a reallocation staff to those clinical areas.

Also, the increased anxiety of the general public after the declaration of the lockdown, led to a significant reduction of attendance to the hospitals, including the emergency services.

Compared with the prepandemic period national data for aortovascular procedures, it seems that the service provision for emergency aortovascular conditions has been protected during the early period of the COVID‐19 pandemic and similar outcomes to pre‐pandemic times have been achieved^13^ (Table 3 and Figures 3, 4).

Overall, the majority of individual centers have been able to maintain similar activity for emergency procedures compared with the prepandemic period, although certain regions more affected by the COVID‐19 disease (i.e., Birmingham) have seen their ability to provide aortovascular cover canceled due to reallocation of the intensive care areas to treat respiratory patients (Figure 2; Table 3).

The impact of not treating aortic diseases has been already reported and a risk of increased 6 months mortality has been recognized.^12^ Therefore, the effect of suspension of elective surgery for patients on waiting lists will be appreciated in months to come.

The preoperative COVID‐19 screening protocol,^9^, ^10^ including the two negative nasopharyngeal swabs and the CT chest without contrast, was implemented at Barts Heart Centre on the 26th March, while other centers implemented it in the following weeks being consistently established by the month of April. However, in the majority of emergency cases, the swab results were not awaited and the operations were carried out providing that the patient did not show any COVID‐19 disease‐related symptoms, weighing in the decision the risk of developing COVID‐19 in the immediate postoperative period against the mortality related to the aortovascular condition.

With this strategy, we have observed a low in‐hospital surgical mortality even for those who were diagnosed of COVID‐19 during the peri‐operative period.

The fact that the majority of patients have been isolating at home during the lockdown period and have been shielded in the hospital only in contact with staff wearing full PPE measures, might have contributed to the low rate of COVID‐19 infection compared with other surgical series. Therefore, this finding of favorable outcomes following aortic surgery during early months of pandemic with appropriate screening protocol and postoperative care with staff wearing full PPE, supports the continuing service provision in subsequent weaves of pandemic. The knowledge of outcomes would be invaluable in consenting patients for surgery during the rest of the pandemic.^14^, ^15^, ^16^, ^17^, ^18^, ^19^

## 5 CONCLUSIONS

The service provision for aortovascular pathologies has changed during the early months of the pandemic while maintaining urgent and emergency activity.

The preoperative COVID‐19 screening protocol, combined with self‐isolation and shielding, contributed to the low incidence of COVID‐19 in our series. Outcomes of surgery for aortovascular patients during this period are comparable with pre‐pandemic national benchmarked results. These results support continuing surgery for this patient group during the recovery phase or future waves of the COVID‐19 pandemic.

## HUMAN STUDIES

This study is registered as service evaluation project and therefore need for ethical approval was not required

## CONFLICT OF INTERESTS

All the authors declare that there are no conflict of interests.

## Data Availability

All data reported in the studies are correct and all authors have equal responsibilities
